# Desmearing small-angle scattering data by central moment expansions of instrument resolution

**DOI:** 10.1107/S1600576725004212

**Published:** 2025-06-20

**Authors:** Guan-Rong Huang, Lionel Porcar, Yuya Shinohara, Christoph U. Wildgruber, Chi-Huan Tung, Changwoo Do, Wei-Ren Chen

**Affiliations:** a Department of Engineering and System Science National Tsing Hua University Hsinchu 30013 Taiwan; b Physics Division, National Center for Theoretical Sciences, Taipei 10617, Taiwan; c Institut Laue–Langevin, BP 156, F-38042 Grenoble Cedex 9, France; d Materials Science and Technology Division Oak Ridge National Laboratory Oak Ridge TN 37831 USA; e Neutron Technologies Division Oak Ridge National Laboratory Oak Ridge TN 37831 USA; f Neutron Scattering Division Oak Ridge National Laboratory Oak Ridge TN 37831 USA; Australian Centre for Neutron Scattering, ANSTO, Australia

**Keywords:** small-angle scattering, desmearing, central moment expansions, instrument resolution effect, soft matter

## Abstract

This study introduces an enhanced numerical technique tailored specifically for refining 1D small-angle scattering (SAS) intensity profiles affected by smearing. Our primary objective is to address the resolution blurring commonly encountered in SAS data, particularly in systems with clearly defined correlation peaks whose spread aligns with the width of the resolution function at corresponding *Q* positions. Unlike previous approaches that expanded the SAS intensity using central moment expansion, the new method focuses on expanding the resolution function itself, thus eliminating artificial oscillations observed in smeared spectra due to limitations inherent in our earlier algorithm. This method is straightforward to implement, computationally efficient and consistently performs well in numerical benchmarking. To illustrate its effectiveness, we present a case study of a lamellar phase characterized by distinct peaks in its small-angle neutron scattering intensities.

## Abstract 2

A strategy based on the central moment expansion approach is proposed for addressing the resolution discrepancy inherent in cross-sectional measurements obtained via small-angle scattering experiments.

## 1. Introduction

The intensity profiles of small-angle scattering (SAS) of neutrons and X-rays are smeared by the finite resolution of instruments, such as the finite beam size and finite angular spread. The smearing often hinders quantitative structural analyses. To obtain smearing-free data from radially averaged, 1D SAS data *I*(*Q*), various desmearing approaches have been developed; these include polynomial approximations, spline interpolations (Taylor & Schmidt, 1967 ▸; Schelten & Hossfeld, 1971 ▸), basis expansion approaches (Hossfeld, 1968 ▸; Vonk, 1971 ▸), regularization techniques (Svergun *et al.*, 1988 ▸) and 2D fast Fourier transform methods (Jaksch *et al.*, 2021 ▸).

We have recently introduced a numerical algorithm rooted in the mathematical framework of central moment expansion (CME) (Huang *et al.*, 2023 ▸). This approach is mathematically equivalent to the unfolding series (Sauder, 1966 ▸). Through computational benchmarking, we have demonstrated the effectiveness of this approach in maintaining numerical consistency in desmeared data when compared with existing deconvolution techniques. Our method has been integrated into the *GRASP* package (Dewhurst, 2023 ▸) for the analysis and reduction of small-angle neutron scattering (SANS) data acquired from instruments at the Institut Laue–Langevin (ILL).

After thorough testing of our methodology, it became apparent that the CME approach introduces spurious features into the desmeared *I*(*Q*). Specifically, when applied to deconvolute experimentally measured *I*(*Q*) from highly ordered systems exhibiting sharp correlation peaks, artificial oscillations manifest alongside these peaks, as detailed below. These artificial features result from the sharpness of the correlation peaks, whose width is comparable to that of the instrumental resolution function.

The objective of this report is to address this issue by modifying the target function for expansion. We propose expanding the resolution function around the sharp peak of the experimentally measured intensities, rather than expanding the ground-truth intensity. This adjustment effectively mitigates the aforementioned problem. We have validated the numerical accuracy of this approach through computational benchmarking and confirmed its practical viability by desmearing the SANS intensity of aqueous solutions of Aerosol OT, a well studied lamellar system (Petrov *et al.*, 2002 ▸) characterized by multiple distinct correlation peaks in *I*(*Q*).

In the following section, we offer a detailed exposition of the proposed methodology, elucidating its mathematical aspects.

## 2. Method

The experimentally measured scattering intensity profile, 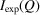, is expressed as 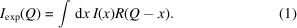
Here, *I*(*Q*) represents the scattering cross section without instrument smearing, and *R*(*Q*) is the instrument resolution function.

**Figure 1:**
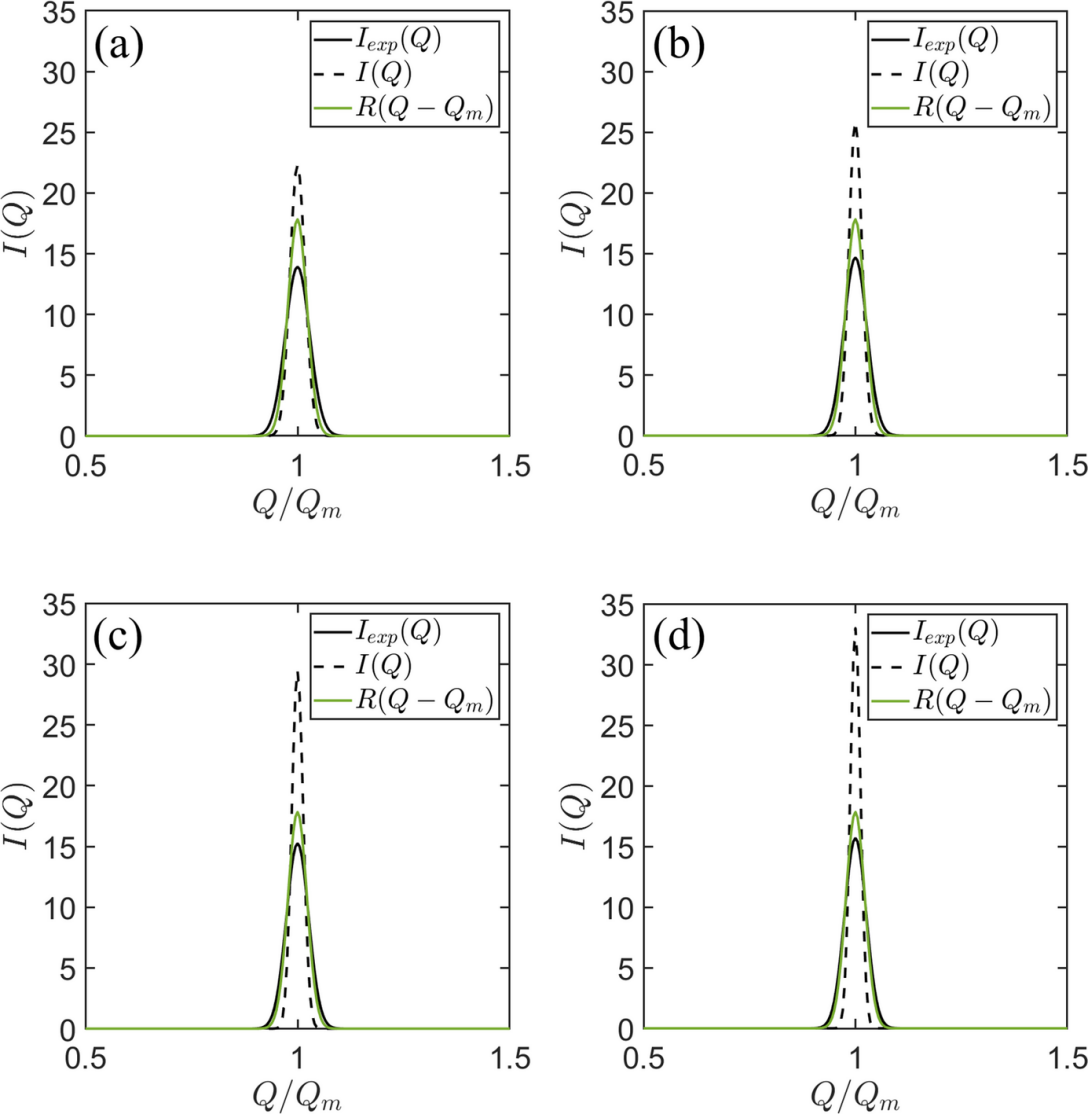
A comparison of the smeared 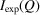 (depicted by black curves), obtained through the convolution of resolution-free scattering intensity *I*(*Q*) (represented by black dashed curves) and resolution function 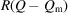 (shown as green curves), characterized by the peak width 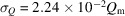. Here, 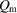 represents the maximum position of 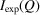, and the peak widths of *I*(*Q*) are 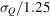, 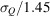, 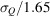 and 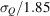 in panels (*a*), (*b*), (*c*) and (*d*), respectively.

Equation (1) indicates that, as *I*(*Q*) approaches a Dirac delta function, 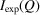 tends to approximate 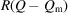. In Fig. 1 ▸, the green line represents the fixed resolution 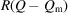, where 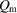 denotes the peak position of 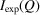. Enhancing the sharpness of 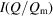, shown by the black dashed line, leads to 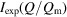 (illustrated by the black solid line) progressively resembling 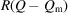.

**Figure 2:**
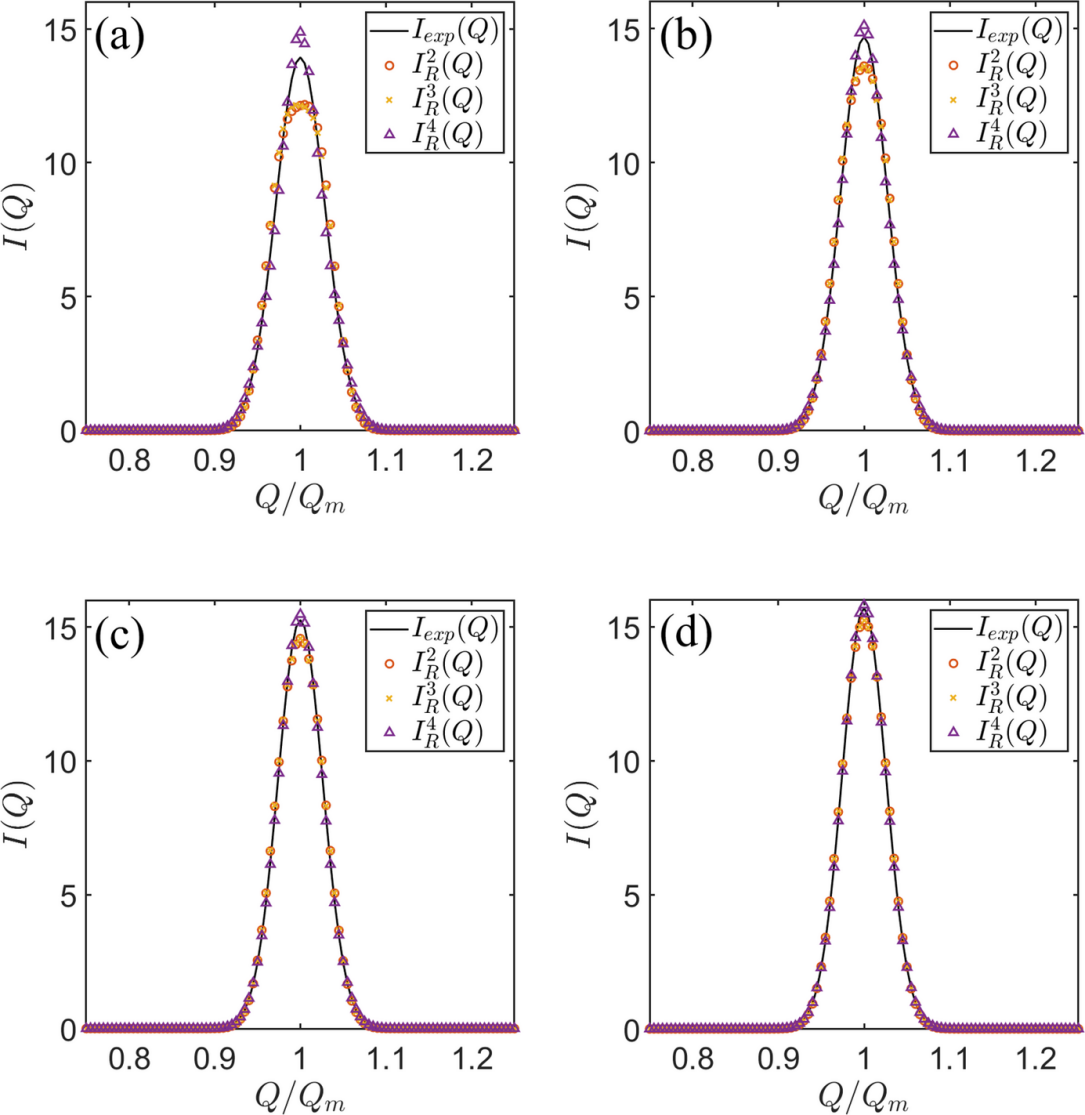
A comparison of smeared 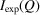 (shown as black curves), two-term CME 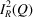 (represented by circles), three-term CME 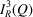 (depicted as crosses) and four-term CME 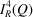 (marked by triangles) for the corresponding *I*(*Q*) and 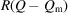 in Fig. 1 ▸.

Therefore, if 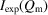 possesses a peak width that is sufficiently small compared with that of 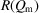, expanding the instrument resolution function by CME at 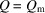 results in the following expression: 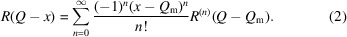
Substituting equation (2) into equation (1) produces a linear combination of central moments of 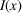 around 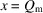: 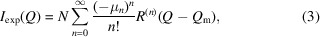
where 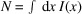 and 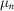 represents the *n*th central moment defined by the following expression: 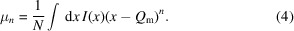
In Fig. 2 ▸, we compare 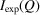 with the summation of various terms outlined in equation (3), where 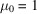. Here, 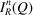 denotes the outcome of summing the initial *n* terms, with the subscript *R* signifying that the CME is conducted over the resolution function *R*(*Q*). When *I*(*Q*) is sufficiently sharp, the first four central moments – mean, variance, skewness and kurtosis – are typically sufficient to reconstruct 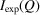. In such cases, the maximum of *I*(*Q*) is highly localized, and contributions from its long-tail distribution are negligible. Therefore, higher-order terms in the CME of equation (1) can be omitted without compromising accuracy. For broader scattering profiles, where the variation in *I*(*Q*) with respect to *Q* is slower than that of the resolution function *R*(*Q*), the inclusion of higher-order terms becomes necessary. Under these conditions, the previous CME method (Huang *et al.*, 2023 ▸), which expands in terms of the central moments of *R*(*Q*), is expected to yield more accurate desmearing results. According to this rationale, 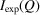 can be expressed as
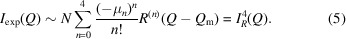
Equation (5) provides the mathematical framework for implementing the desmearing of 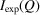. The initial step involves performing regression analysis on 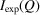 using equation (5) to determine the numerical values of *N* and 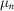. Extracted values of *N* and 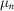 are used as inputs in equation (4) to reconstruct *I*(*Q*) via the principle of maximum probabilistic entropy (Kardar, 2007 ▸). In this approach, the information entropy *S* is defined by the following integral: 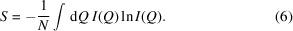
Subject to the constraint outlined in equation (4), one viable strategy for maximizing *S* involves using the method of Lagrange multipliers (Wylie & Barrett, 1995 ▸). This approach yields the following analytical equations for *I*(*Q*): 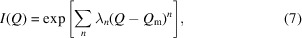
where 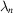 are constants and can be determined by equation (4). Using the maximum probabilistic entropy approach with the first three central moments results in a Gaussian function. Moreover, incorporating higher-order central moments effectively addresses the skewness and kurtosis of *I*(*Q*). The key difference between the present method and our previous approach (Huang *et al.*, 2023 ▸) lies in the choice of the expansion function from equation (1) to equation (7). Specifically, the current method expands equation (1) using the central moments of the scattering intensity, whereas the previous method employed the central moments of the resolution function *R*(*Q*). This modification is more suitable when the scattering profile is localized with insignificant long tails and sharper than the resolution function. Moreover, the present method exhibits reduced sensitivity to noise amplification, as it avoids the use of the second derivative of the experimentally measured *I*(*Q*) required in the previous approach. While the two formulations are mathematically equivalent, their applicability depends on the relative sharpness of the scattering intensity and the resolution function.

**Figure 3:**
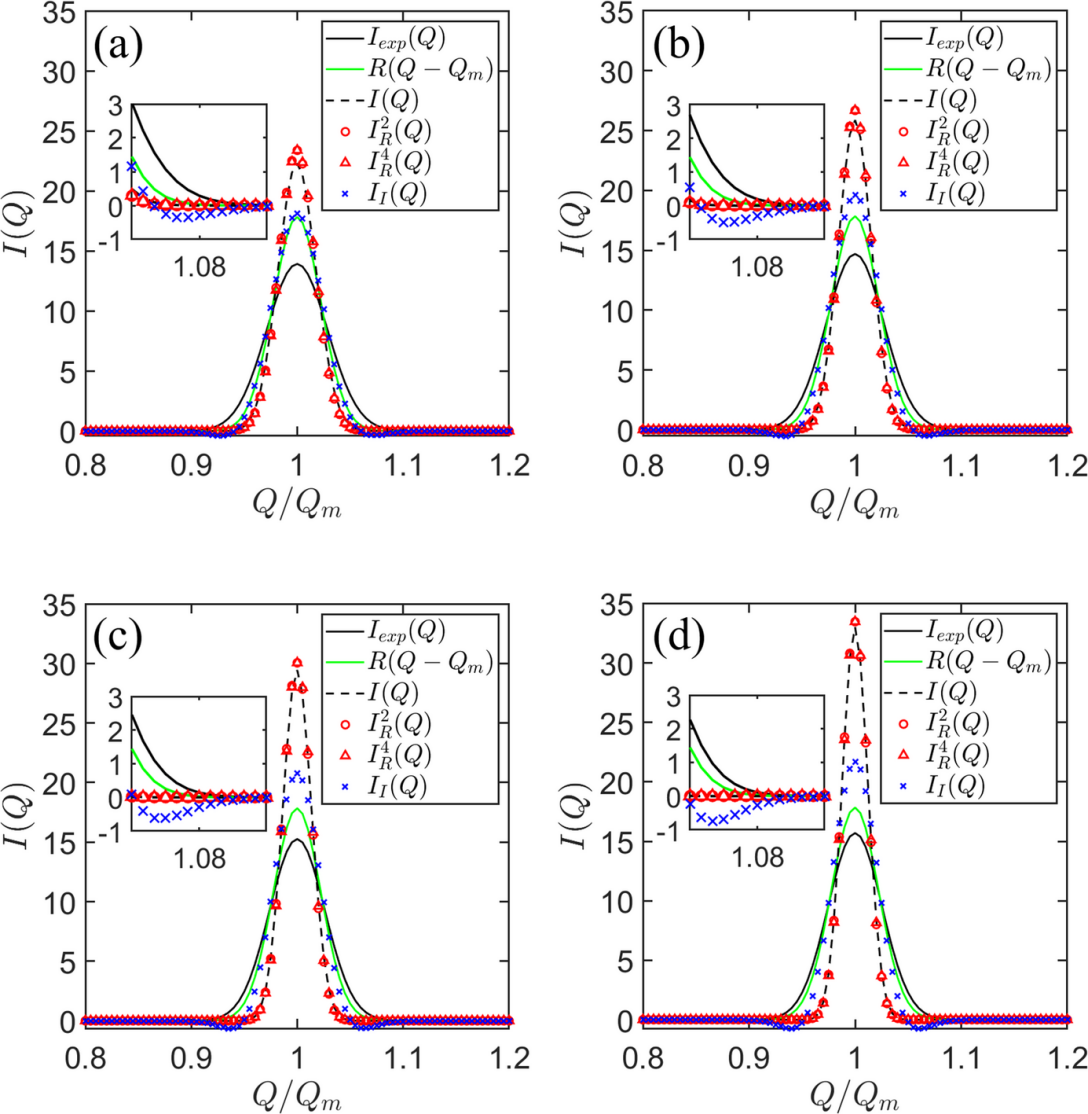
A comparison of various scattering intensities: smeared 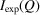 (depicted by black curves), resolution function (green curves), resolution-free scattering intensity *I*(*Q*) (shown as black dashed curves), and desmeared intensities 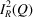 (indicated by red circles) and 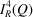 (represented by red triangles), using central moment values extracted in Fig. 2 ▸. 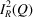 and 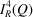 denote the reconstructed scattering intensities using the maximum entropy approach with two- and four-term central moments. Additionally, 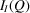 (marked as blue crosses) represents the reconstructed scattering intensity obtained from our previously reported desmeared algorithm (Huang *et al.*, 2023 ▸). The insets in panels (*a*)–(*d*) show magnified views of the scattering profiles near the inter-particle correlation peaks, providing visual evidence of artificial oscillation behavior.

A numerical benchmarking was conducted to assess the feasibility of the desmearing algorithm that combines CME and maximum entropy, as shown in Fig. 3 ▸. When employing the same resolution function 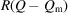, the scattering intensity obtained from the CME of *I*(*Q*), denoted as 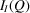, exhibits artificial oscillations around both sides of the correlation peaks at 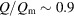 and 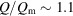. Additionally, the desmeared correlation peak appears less well defined, with 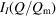 observed to be lower and the width broader compared with 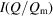. We identified that the cause of this numerical artifact is the faster rate of change of *I*(*Q*) with respect to *Q*, compared with that of *R*(*Q*).

## 3. Assessing the feasibility of desmearing experimental data

To validate the viability of our proposed desmearing method, we conducted SANS on a well studied lyotropic system: sodium dioctyl sulfosuccinate, commonly known as AOT, sourced from Thermo Scientific. Aqueous solutions of AOT with varying weight concentrations of 40% and 50% were prepared by dissolving AOT powders in deuterium oxide (D_2_O) procured from Sigma Aldrich, ensuring a deuteration degree of at least 99.9%. These solutions were continuously stirred under standard environmental conditions for approximately 4 h, resulting in optically transparent solutions. Notably, the concentration range investigated has been previously identified as equilibrium lamellar phases (Petrov *et al.*, 2002 ▸).

The SANS experiments were performed using the D22 large-dynamic-range small-angle diffractometer at the ILL, with the following instrument parameters: beam size of 7 mm × 10 mm, collimation length of 17.6 m, and triangular-shaped pixel dimensions of 8 mm × 4 mm along the horizontal and vertical directions, respectively. To achieve comprehensive coverage of the essential *Q* range from 0.001 to 0.5 Å^−1^, where coherent neutron scattering was observed, two wavelengths of 6 and 11.5 Å, with a wavelength spread of 10% FWHM, were used. The AOT aqueous solutions were contained in Hellma banjo cells with a path length of 1 mm. The measurements were carried out at two temperatures of 50 and 80°C.

**Figure 4:**
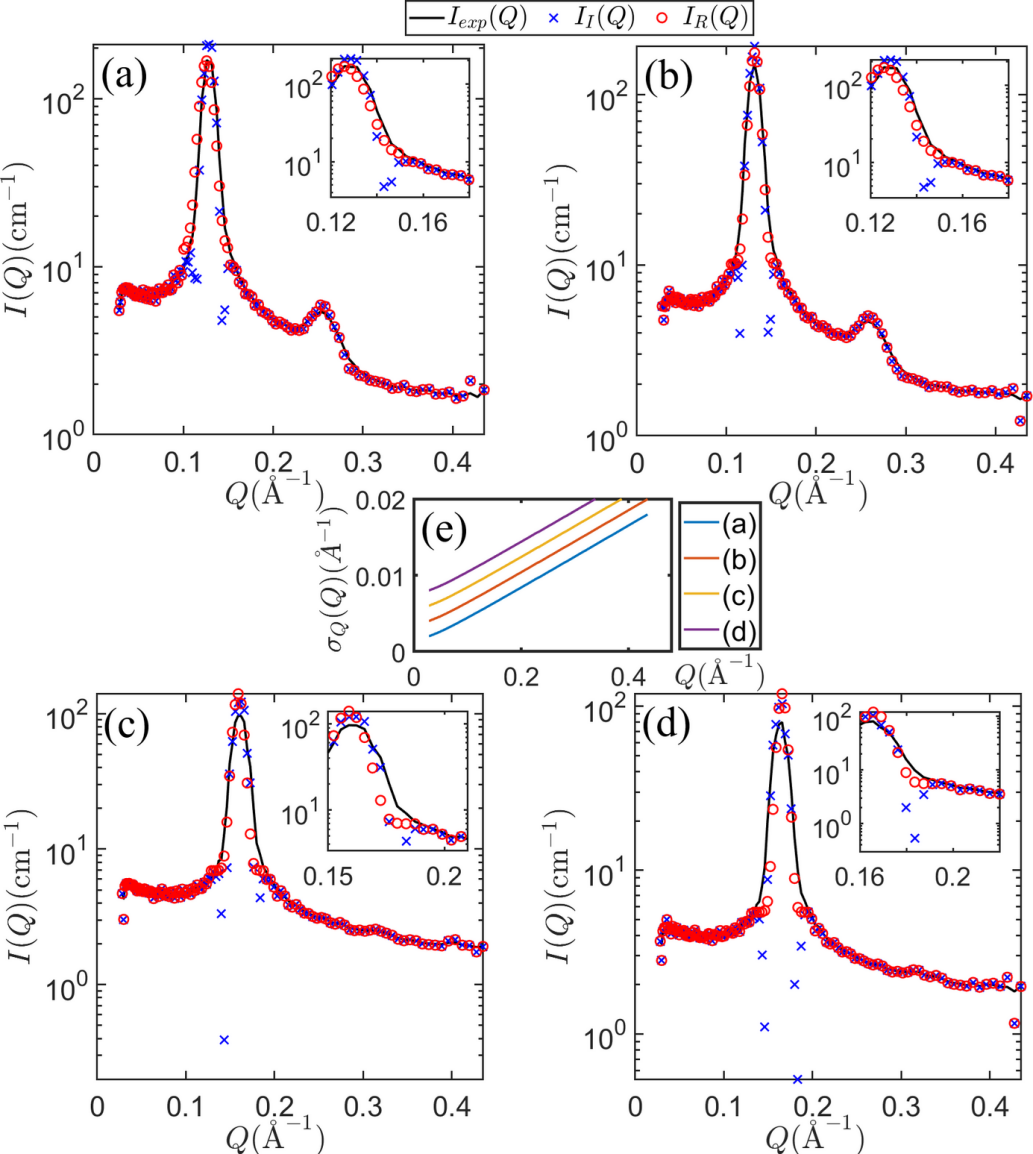
The SANS intensities of AOT molecules immersed in fully deuterated water with concentration 40 wt% at 50°C (*a*) and 80°C (*b*), and with concentration 50 wt% at 50°C (*c*) and 80°C (*d*). Panel (*e*) shows the standard deviation 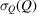 of the resolution function *R*(*Q*) as a function of *Q* for the cases presented in panels (*a*)–(*d*). Since the 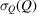 curves are nearly identical, the values corresponding to panels (*b*), (*c*) and (*d*) are vertically offset by 0.002, 0.004 and 0.006 Å^−1^, respectively, to facilitate visual comparison. The insets in panels (*a*)–(*d*) show magnified views of the scattering profiles near the inter-particle correlation peaks, providing visual evidence of artificial oscillation behavior. 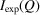 represents the experimentally measured scattering intensity, 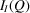 stands for the corresponding desmeared data using the previously developed CME method, and 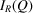 is the desmeared intensity using the maximum entropy approach with four-term central moments. For *Q* values outside the range of 0.15 to 0.2 Å^−1^, 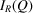 is set to be equal to 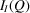.

Fig. 4 ▸ presents the experimentally measured SANS intensity 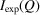 (depicted by black curves), juxtaposed with 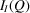 (represented by blue symbols) and 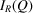 (represented by red symbols). Notably, the artificial oscillations previously reported in 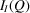 (Tung *et al.*, 2024 ▸) are no longer discernible in 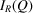. Moreover, except for the *Q* range from 0.15 to 0.2 Å^−1^, where the peak width of *I*(*Q*) is comparable to that of *R*(*Q*), 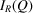 is set to be identical to 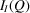. This decision is informed by the small standard deviation of *R*(*Q*) compared with that of *I*(*Q*), and the absence of artificial oscillations in 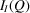.

## 4. Conclusion

This study introduces a numerical algorithm designed to address the impact of instrument resolution on SANS data. It represents a conceptual extension of our previously developed desmearing algorithm (Huang *et al.*, 2023 ▸). Both algorithms utilize the central moment expansion scheme to extract relevant parameters, albeit from different target functions. In particular, the present method focuses on scenarios where correlation peaks exhibit a width comparable with that of the instrument resolution function. Computational benchmarking confirms the numerical accuracy of the approach, and its practical feasibility is demonstrated through application to a series of SANS intensity profiles from lamellar solutions featuring sharp correlation peaks.

Looking ahead, the proposed framework is mathematically extendable to the desmearing of higher-dimensional scattering data by applying the central moment expansion along multiple directions in reciprocal space. Moreover, the method is well suited for integration into widely used small-angle scattering analysis platforms, such as *SASView* (https://www.sasview.org/),*GRASP* (ILL) and user environments like the *Galaxy* platform (https://galaxyproject.org/) used at Oak Ridge National Laboratory. Such integration could enhance the accessibility of the method and promote its broader adoption within the small-angle scattering community.
